# Oncogenic transformation of murine C3H 10T1/2 cells resulting from DNA double-strand breaks induced by a restriction endonuclease.

**DOI:** 10.1038/bjc.1989.378

**Published:** 1989-12

**Authors:** P. E. Bryant, A. C. Riches

**Affiliations:** Department of Biology and Preclinical Medicine, University of St Andrews, Fife, UK.

## Abstract

**Images:**


Br. J. Cancer (1989), 60, 852-854 © The Macmillan Press Ltd., 1989

SHORT COMMUNICATION

### Oncogenic transformation of murine C3H 10T1/2 cells resulting from DNA double-strand breaks induced by a restriction endonuclease

P.E. Bryant & A.C. Riches

*Department of Biology and Preclinical Medicine, University of St Andrews, St Andrews, Fife KY16 5TS, UK.*

Ionising radiation has been acknowledged as an effective carcinogen for several decades but the nature of the primary lesions in the DNA of exposed cells which cause them to undergo oncogenic transformation is not known. It is generally agreed that oncogenic transformation is a multistage process (Barrett & Fletcher, 1987) at the very least requiring the interaction of two events: an 'initiation' and a 'promotion' event. These may take the form of, for example, the activation or abnormal expression of more than one oncogene, such as c-*ras* and c-*myc* (e.g. Balmain & Pragnell, 1983; Balmain, 1985), or the activation of a mutated oncogene (Burck *et al.*, 1988; Bradshaw, 1986). For *in vivo* systems the complex nature of the process is indicated by the multiplicity of factors influencing the outcome of initial treatments (e.g. Fry, 1981; Upton, 1984). Although the essential nature of the transformation process is not yet understood it is a frequent observation that karyotypic changes, particularly translocations, have taken place in transformed cells (e.g. the Philadelphia chromosome in chronic myeloid leukaemia) which may be causally related to the transformation process (Klein & Klein, 1984). Oncogenic transformation of cultured cells can be studied in the murine C3H 10T1/2 system (Reznikoff *et al.*, 1973; Han & Elkind, 1979; Kennedy *et al.*, 1980). The 10T1/2 system may not be an ideal system as a model for the study of cancer induction in the whole animal. However, it is one of the few test systems available for agents or factors which are known carcinogens in animals or humans. Transformation of 10T1/2 cells from the 'normal' cell phenotype to foci of uncontrolled cell growth allows the quantification of the effects of radiation or other genotoxic agents. These foci of rapid growth (type III foci) can be shown to yield malignant tumours in syngeneic mice after subcutaneous inoculation of a sufficient number of cells.

Although it has been possible to obtain dose-effect relationships for induction of transformed foci as a function of radiation dose, the mechanisms of induction of these by radiation are not yet understood. In particular it has not been possible to identify the primary lesion or lesions responsible for the genetic alterations of transformed cells.

One of the main problems is that ionising radiation induces several different types of initial lesions in the DNA of exposed cells. These are direct DNA strand breaks (single or double), base lesions, and cross-links between strands of DNA or between DNA and protein. For several radiation-damage end-points, in eukaryotic cells (e.g. cell death, chromosome aberrations and mutations) the DNA double-strand break (dsb) has been implicated as the causative lesion (Frankenberg *et al.*, 1984; Natarajan *et al.*, 1980). Moreover, it has been shown that exposure of permeabilised mammalian cells to type II restriction endonucleases (RE) which induce double-strand breaks in DNA at specific recognition sequences leads to the induction of chromosomal aberrations (for a review see Bryant, 1988). It was shown previously that RE, which induce 'blunt-ended' dsb, were more effective than those inducing cohesive-ended dsb (Bryant, 1984). In these studies entry of RE was facilitated by the simultaneous exposure of Chinese hamster cells to inactivated Sendai virus and RE. Murine cells were also shown to be susceptible to this treatment (Natarajan & Obe, 1984), and in both hamster and mouse cells aberrations were observed of the same types as those induced by X-rays, including both exchanges and deletions (Bryant, 1984; Natarajan & Obe, 1984). On the basis of the observation that visible chromosomal alterations are often associated with cancers in man and other mammals (Klein & Klein, 1984), it seemed plausible that an initiating lesion in radiogenic cancers might be the DNA dsb. In addition it has been observed that restriction endonuclease induced dsb lead to mutations at the hgprt locus in Chinese hamster cells (Obe *et al.*, 1986). Mutations were not induced at the $NA^+/K^+$ ATPase locus by the same treatment, and the reason is thought to be that this type of genetic change involves a point mutation rather than a 'chromosomal' mutation as in the case of the hgprt locus.

In the light of the foregoing evidence we have examined the possibility that oncogenic transformation can be induced by treatment of Sendai virus permeabilised 10T1/2 cells with the restriction endonuclease Pvu II which induces blunt-ended dsb at the sequence CAG'GTC; a procedure known to lead to induction of chromosomal aberrations in hamster cells (Bryant, 1984).

Low passage C3H10T1/2 cells were cultured in basal Eagle's medium with addition of 10% fetal calf serum (BMEFCS). Cells were seeded into 75 $cm^2$ flasks at approximately $10^5$ cells per flask and grown for 3 days, when they were sub-confluent. From this culture, 25 $cm^2$ experimental flasks were seeded from this culture at $2 \times 10^5$ cells per flask, and these were incubated for a further 3 days, so that cells were still sub-confluent. For enzyme treatments, medium was aspirated from flasks and the cell layers rinsed once with Hank's balanced salts solution (HBSS) containing 1% bovine serum albumin and 6 mmol $l^{-1}$ $MgCl_2$. This solution was aspirated and 300 µl of ice-cold UV-inactivated Sendai virus (ISV), containing approximately 1200 HAU $ml^{-1}$, was added together with various amounts of Pvu II and flasks placed on ice for 10 min. After this, flasks were transferred to an incubator at 37°C for 30 min. During incubations flasks were tilted from side to side at intervals to ensure that the virus/enzyme mixture was evenly dispersed over the cell layer. After treatment the liquid was aspirated and cells rinsed once with 2 ml of warm BMEFCS and 5 ml of BMEFCS added to each flask and placed in an incubator at 37°C for 3-4 h.

Both colony forming ability and transformation assays were performed on each treated flask. Cells were trypsinised and counted. For the colony assay approximately 100 viable cells were plated in each of four 10 cm dishes with 10 ml BMEFCS and incubated for 10-14 days. For the transformation assay, 300 surviving cells were plated in each of 30 flasks (75 $cm^2$) and incubated for 8 weeks with weekly medium changes. After cells became confluent the serum content was reduced to 5%. Medium was aspirated and cells rinsed in Sorensen's buffer (pH 6.4) and then cells were fixed

Correspondence: P.E. Bryant.
Received 20 March 1989; and in revised form 10 July 1989.

in methanol for 10 min. Cells were finally stained in 3% Giemsa for 30 min, rinsed with buffer and allowed to dry.

Scoring of transformed foci was carried out according to the classification of Reznikoff *et al.* (1973). Mean frequencies of transformed foci were determined from the frequency of negative flasks by assuming a Poisson distribution according to the method of Han and Elkind (1979).

The same types of transformed foci were observed as those induced by ionising radiation and these were classified into types I, II and III. An example of a Pvu II induced focus is shown in Figure 1, illustrating the characteristic multilayering and criss-cross patterning. The combined frequency of type II and III foci was determined in control (untreated), enzyme storage buffer and virus treated (SB) and Pvu II treated samples. The results are presented in Tables I and II. These show that a significantly higher number of transformed foci were observed in samples of cells treated at the highest enzyme dose (300 U ml$^{-1}$). Although the increase in transformation frequency was small in the enzyme treated group it was significantly different from the storage buffer and inactivated virus treated (ISV + SB) control. Using a $\chi^2$ test (Parker, 1979), comparing the number of positive flasks in the Pvu II treated with the number of positive flasks in the ISV + SB group, there was a significant difference ($\chi^2$ correlation = 4.70, $P<0.05$; Table I). A similar conclusion is reached by calculating the standardised normal deviate ($d = 2.00$, $P<0.05$) for the two Poisson counts (Parker, 1979). Comparing the results of the transformation frequency

**Table I** Frequencies of positive (presence of type II or III foci) and negative flasks in Pvu II treated samples

| *Treatment* | *No. + ve flasks* | *No. − ve flasks* | *Total no. flasks* | *Fraction flasks − ve (F)* | *Average transformants/ flask (ATF)* |
|---|---|---|---|---|---|
| Control | 13 | 106 | 119 | 0.891 | 0.1157 |
| ISV + SB | 11 | 109 | 120 | 0.908 | 0.0961 |
| ISV + 150 (U ml$^{-1}$) | 13 | 104 | 117 | 0.889 | 0.1178 |
| ISV + 300 (U ml$^{-1}$) | 24 | 97 | 121 | 0.802 | 0.2211 |

Pvu II concentration is given in U ml$^{-1}$. Pooled results from four independent experiments. SB, storage buffer; ISV, inactivated Sendai virus; F, fraction of flasks with no transformed foci; ATF, average number of transformants per flask calculated from ($-\ln_e$ F) assuming Poisson statistics (Han & Elkind, 1979).

(TF1; Table II) for individual experiments using a $t$ test also suggests there is a significant difference ($t = 2.29$, $P<0.05$; Table II).

**Table II** Transformation frequencies per viable cell (TF) calculated from data in Table I

| *Treatment* | *TF 1 (× 10$^{-4}$)* | *TF 2 (× 10$^{-4}$)* |
|---|---|---|
| Control | 3.56 ± 1.34 | 3.86 |
| ISV + SB | 3.11 ± 1.36 | 3.20 |
| ISV + 150 (U ml$^{-1}$) | 3.52 ± 1.10 | 3.93 |
| ISV + 300 (U ml$^{-1}$) | 7.16 ± 1.14 | 7.37 |

Pvu II concentration is given in U ml$^{-1}$. Pooled results from four independent experiments. TF1, transforming frequency calculated from the observed number of transformants per flask (mean ± s.e.; four separate experiments); TF2, transforming frequency calculated from the number of negative flasks (see Table I); SB, storage buffer; ISV, inactivated Sendai virus.

At the highest enzyme dose used (300 U ml$^{-1}$) there was only a small effect on cell survival (surviving fraction = 0.91 ± 0.09) compared to untreated controls. The surviving fraction for the ISV + SB group was 0.94 ± 0.06. It is pertinent to note that 10T1/2 cells exhibit a prominent shoulder on the survival curve following X-ray treatment. A similar transformation frequency has been observed at radiation doses of about 2 Gy (Han & Elkind, 1979; Kolman *et al.*, 1989; Terzaghi & Little, 1976) where the cell survival is also only slightly decreased from controls (surviving fraction = 0.8–0.9).

The results thus show a significantly higher frequency of transformation in samples treated with Pvu II at 300 U ml$^{-1}$ than in control or storage-buffer treated groups. We infer from this result that one of the events in the oncogenic transformation of cells may be one or more DNA dsb. The mechanism by which dsb might be involved in oncogenic transformation is not known but could entail non-repair or misrepair (e.g. translocation) of dsb. Both chromosomal aberrations of exchange and deletion types, and mutations at the hgprt locus have been shown to arise in restriction endonuclease treated cells (Bryant, 1984; Obe *et al.*, 1986). It is therefore possible that the oncogenic transformation of 10T1/2 cells could result from such cytogenetic alterations or via mutation. Experiments to determine the frequency of chromosomal aberrations induced by Pvu II and other restriction enzymes, e.g. those producing cohesive rather than blunt-ended dsb in 10T1/2 cells, are in progress. The nature of the oncogenic change involved in

**Figure 1** The edge of a type III focus induced by Pvu II, showing the multilayering and 'criss-cross' appearance at the edges of the focus. This contrasts with the normal monolayer formed at confluence by 10T1/2 cells, which can be seen outside the focus.

transformation of 10T1/2 cells is not yet clear. The oncogene c-*myc* is known to be expressed in transformed foci. However, the presence of a mutated c-*ras* gene has not been demonstrated so far (Sawey *et al.*, 1988). Borek *et al.* (1987) have demonstrated the presence of a unique non-ras transforming gene in cell lines derived from irradiated hamster embryo cells and mouse C3H 10 T1/2 cells.

This work was supported by a grant from the Medical Research Council.

### References

BALMAIN, A. (1985). Transforming ras oncogenes and multistage carcinogenesis. *Br. J. Cancer*, **51**, 1.

BALMAIN, A. & PRAGNELL, I.B. (1983). Mouse skin carcinomas induced in vivo by chemical carcinogens have a transforming Harvey-*ras* oncogene. *Nature*, **303**, 72.

BARRETT, J.C. & FLETCHER, W.F. (1987). Cellular and molecular mechanisms of multistep carcinogenesis in cell culture models. In *Mechanisms of Environmental Carcinogenesis: Volume 2: Multistep Models of Carcinogenesis*, Barrett, J.C. (ed.) p. 73. CRC Press: Boca Raton.

BOREK, C., ONG, A. & MASON, H. (1987) Distinctive transforming genes in X-ray-transformed mammalian cells. *Proc. Natl Acad. Sci. USA*, **84**, 794.

BRADSHAW, T.K. (1986). Cell transformation: the role of oncogenes and growth factors. *Mutagenesis*, **1**, 91.

BRYANT, P.E. (1984). Enzymatic restriction of mammalian cell DNA using Pvu II and Bam H1: evidence for the double-strand break origin of chromsonal aberrations. *Int. J. Radiat. Biol.*, **46**, 57.

BRYANT, P.E. (1988). Use of restriction endonucleases to study relationships between DNA double-strand breaks, chromosomal aberrations and other end-points in mammalian cells. *Int. J. Radiat. Biol.*, **54**, 869.

BURCK, K.B., LIN, E.T. & LARRICK, J.W. (1988). *Oncogenes–an Introduction to the Concept of Cancer Genes*. Springer Verlag: New York.

FRANKENBERG, D., FRANKENBERG-SCHWAGER, M. & HARBICH, R. (1984). Interpretation of the shape of survival curves in terms of induction and repair-misrepair of DNA double-strand breaks. *Br. J. Cancer*, **49**, Suppl. VI, 233.

FRY, R.J.M. (1981). Experimental radiation carcinogenesis: what have we learned? *Radiat. Res.*, **87**, 224.

HAN, A. & ELKIND, M. (1979). Transformation of mouse C3H10T1/2 cells by single and fractionated doses of X-rays and fission-spectrum neutrons. *Cancer Res.*, **39**, 123.

KENNEDY, A.R., FOX, M., MURPHY, G. & LITTLE, J.B. (1980). Relationship between X-ray exposure and malignant transformation in C3H10T1/2 cells. *Proc. Natl Acad. Sci. USA*, **77**, 7262.

KLEIN, G. & KLEIN, E. (1984). Oncogene activation and tumour progression. *Carcinogenesis*, **5**, 429.

KOLMAN, A., NASLUND, M., OSTERMAN-GOLKAR, S., SCALIA-TOMBA, G. & MEYER, A. (1989). Comparative studies of in vitro transformation by ethylene oxide and gamma radiation of C3H10T1/2 cells. *Mutagenesis*, **4**, 58.

NATARAJAN, A.T., OBE, G., VAN ZEELAND, A.A., PALITTI, F., MEIJERS, M. & VERDEGAAL-IMMERZEEL, E.A.M. (1980). Molecular mechanisms involved in the production of chromosomal aberrations. II Utilization of Neurospora endonuclease for the study of aberration production by X-rays in G1 and G2 stages of the cell cycle. *Mutation Res.*, **69**, 293.

NATARAJAN, A.T. & OBE, G. (1984). Molecular mechanisms involved in the production of chromosomal aberrations. *Chromosoma*, **90**, 120.

OBE, G., VON DER HUDE, W., SCHEUTWINKEL-REICH, M. & BASLER, A. (1986). The restriction endonuclease Alu I induces chromosomal aberrations and mutations in the hypoxanthine phosphoribosyl transferase locus but not in the $Na^{+}/K^{+}$ ATPase locus in V79 hamster cells. *Mutation Res.*, **174**, 71.

PARKER, R.E. (1979). *Introductory Statistics for Biology*, 2nd edn. Edward Arnold: London.

REZNIKOFF, C.A., BRANKOW, D.W. & HEIDELBERGER, C. (1973). Establishment and characterisation of a cloned line of C3H mouse embryo cells sensitive to post-confluence inhibition of cell division. *Cancer Res.*, **33**, 3231.

SAWEY, M.J. & KENNEDY, A.R. (1989). Activation of oncogenes in radiation induced malignant transformation. In *Biological Basis of Risk Assessment (Proceedings of the 14th L.H. Gray Meeting Oxford, 1988)*, Baverstock, K. (ed.). Taylor & Francis: London.

TERZAGHI, M. & LITTLE, J.B. (1976). X-radiation-induced transformation in a C3H mouse embryo-derived cell line. *Cancer Res.*, **36**, 1367.

UPTON, A.C. (1984). Biological aspects of radiation carcinogenesis. In *Radiation Carcinogenesis: Epidemiology and Biological Significance*, Boice, J.D. & Fraumeni, J.F. (eds) p. 9. Raven: New York.


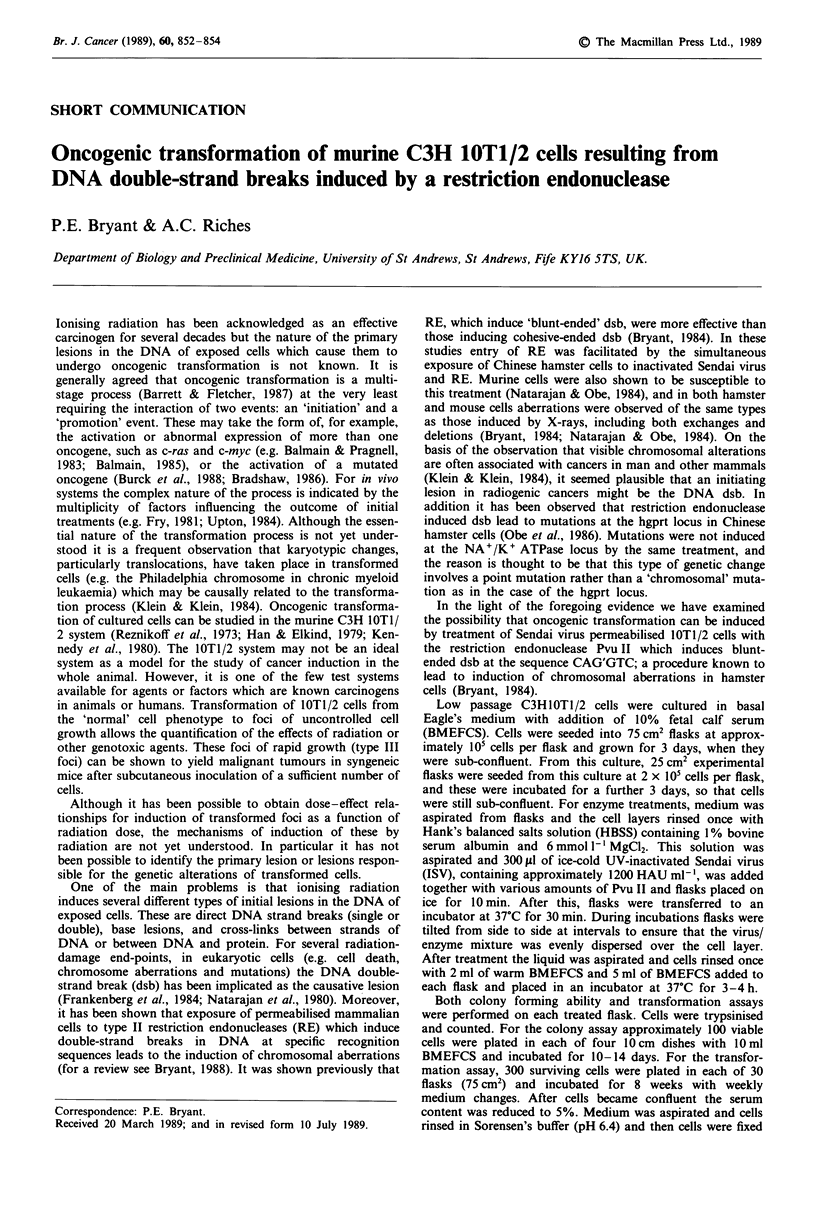

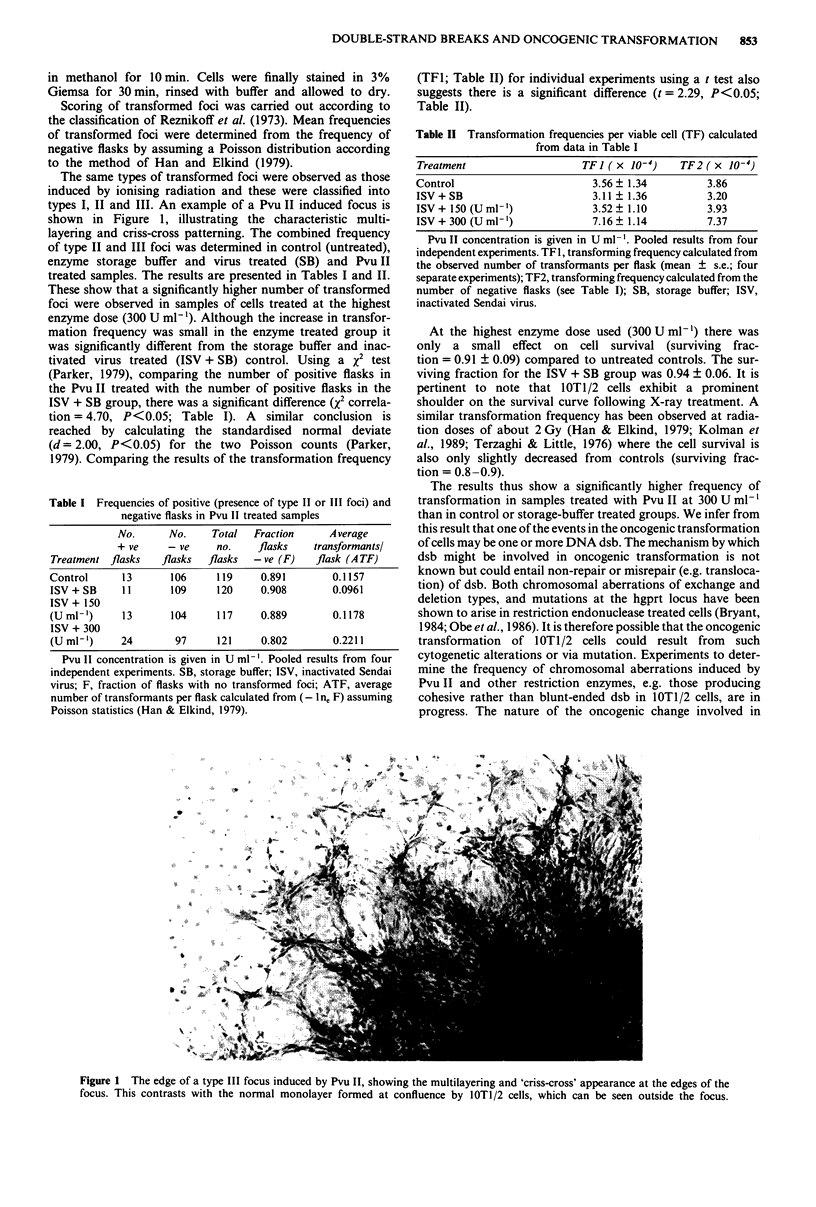

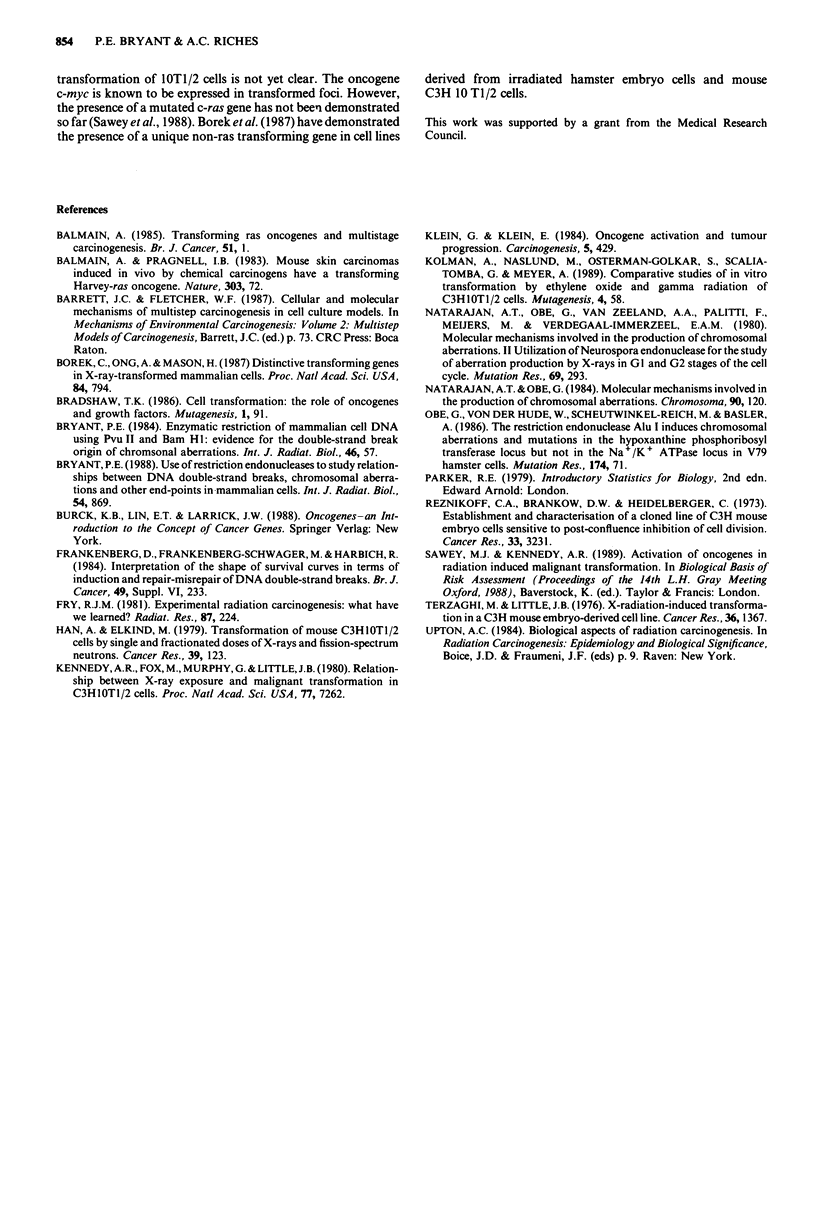


## References

[OCR_00274] Balmain A., Pragnell I. B. (1983). Mouse skin carcinomas induced in vivo by chemical carcinogens have a transforming Harvey-ras oncogene.. Nature.

[OCR_00270] Balmain A. (1985). Transforming ras oncogenes and multistage carcinogenesis.. Br J Cancer.

[OCR_00286] Borek C., Ong A., Mason H. (1987). Distinctive transforming genes in x-ray-transformed mammalian cells.. Proc Natl Acad Sci U S A.

[OCR_00291] Bradshaw T. K. (1986). Cell transformation: the role of oncogenes and growth factors.. Mutagenesis.

[OCR_00295] Bryant P. E. (1984). Enzymatic restriction of mammalian cell DNA using Pvu II and Bam H1: evidence for the double-strand break origin of chromosomal aberrations.. Int J Radiat Biol Relat Stud Phys Chem Med.

[OCR_00300] Bryant P. E. (1988). Use of restriction endonucleases to study relationships between DNA double-strand breaks, chromosomal aberrations and other end-points in mammalian cells.. Int J Radiat Biol.

[OCR_00311] Frankenberg D., Frankenberg-Schwager M., Harbich R. (1984). Interpretation of the shape of survival curves in terms of induction and repair/misrepair of DNA double-strand breaks.. Br J Cancer Suppl.

[OCR_00317] Fry R. J. (1981). Experimental radiation carcinogenesis: what have we learned?. Radiat Res.

[OCR_00321] Han A., Elkind M. M. (1979). Transformation of mouse C3H/10T1/2 cells by single and fractionated doses of X-rays and fission-spectrum neutrons.. Cancer Res.

[OCR_00326] Kennedy A. R., Fox M., Murphy G., Little J. B. (1980). Relationship between x-ray exposure and malignant transformation in C3H 10T1/2 cells.. Proc Natl Acad Sci U S A.

[OCR_00331] Klein G., Klein E. (1984). Oncogene activation and tumor progression.. Carcinogenesis.

[OCR_00337] Kolman A., Näslund M., Osterman-Golkar S., Scalia-Tomba G. P., Meyer A. (1989). Comparative studies of in vitro transformation by ethylene oxide and gamma-radiation of C3H/10T1/2 cells.. Mutagenesis.

[OCR_00349] Natarajan A. T., Obe G. (1984). Molecular mechanisms involved in the production of chromosomal aberrations. III. Restriction endonucleases.. Chromosoma.

[OCR_00341] Natarajan A. T., Obe G., van Zeeland A. A., Palitti F., Meijers M., Verdegaal-Immerzeel E. A. (1980). Molecular mechanisms involved in the production of chromosomal aberrations. II. Utilization of Neurospora endonuclease for the study of aberration production by X-rays in G1 and G2 stages of the cell cycle.. Mutat Res.

[OCR_00352] Obe G., Von der Hude W., Scheutwinkel-Reich M., Basler A. (1986). The restriction endonuclease Alu I induces chromosomal aberrations and mutations in the hypoxanthine phosphoribosyltransferase locus, but not in the Na+/K+ ATPase locus in V79 hamster cells.. Mutat Res.

[OCR_00363] Reznikoff C. A., Brankow D. W., Heidelberger C. (1973). Establishment and characterization of a cloned line of C3H mouse embryo cells sensitive to postconfluence inhibition of division.. Cancer Res.

[OCR_00375] Terzaghi M., Little J. B. (1976). X-radiation-induced transformation in a C3H mouse embryo-derived cell line.. Cancer Res.

